# Variation of Energy in Photobiomodulation for the Control of Radiotherapy-Induced Oral Mucositis: A Clinical Study in Head and Neck Cancer Patients

**DOI:** 10.1155/2018/4579279

**Published:** 2018-02-22

**Authors:** Cizelene do Carmo Faleiros Veloso Guedes, Silas Antonio Juvencio de Freitas Filho, Paulo Rogério de Faria, Adriano Mota Loyola, Robinson Sabino-Silva, Sérgio Vitorino Cardoso

**Affiliations:** ^1^Area of Pathology, School of Dentistry, Federal University of Uberlândia, Uberlândia, MG, Brazil; ^2^Department of Surgery, Stomatology, Pathology, and Radiology, Area of Pathology, Bauru School of Dentistry, University of São Paulo, Bauru, SP, Brazil; ^3^Department of Morphology, Biomedical Sciences Institute, Federal University of Uberlândia, Uberlândia, MG, Brazil; ^4^Department of Physiology, Biomedical Sciences Institute, Federal University of Uberlândia, Uberlândia, MG, Brazil

## Abstract

Oral mucositis (OM) is a frequent and severe adverse effect of therapy against head and neck cancer. Photobiomodulation with the low-power laser is known to be effective against OM, but the diversity of protocols and the possibility of stimulating residual tumor cells are still obstacles. The present study aimed to compare two doses of laser energy delivered to the oral mucosa of patients under oncologic treatment for head and neck cancer, looking for differences in the control of mucositis, as well as in the frequency of tumoral recurrences. Fifty-eight patients undergoing radiotherapy were randomized into two groups, distinguished according to the energy delivered by laser irradiation, namely, 0.25 J and 1.0 J. The groups were compared according to frequency, severity, or duration of OM, as well as the frequency of tumoral recurrences. OM was significantly less frequent in patients receiving 1.0 J of energy, but the groups did not differ regarding severity or duration of OM. Tumoral recurrence also did not vary significantly between the groups. Photobiomodulation with a higher dose of energy (1.0 J versus 0.25 J) is associated with better control of radiotherapy-induced OM and does not significantly increase the risk of neoplastic recurrence.

## 1. Introduction

Oral mucositis (OM) is an acute and ulcerative inflammation of the oropharyngeal mucosa caused by cytotoxic cancer therapy [1]. It is one of the most common adverse effects of head and neck irradiation and is even more frequent when associated with chemotherapy [2]. The course of OM frequently leads to severe pain that is sufficiently severe to impair speech, eating, and swallowing, thus reducing the quality of life of the patients [3]. These events can lead to hospital admission involving substantial additional costs and even interruption of oncologic treatment [4].

The injury to healthy tissue caused by irradiation and OM-related effects begins with cellular death triggered by direct damage to DNA, followed by intense oxidative stress [5]. Most of the injury has been associated with the latter effect, which activates and amplifies signaling pathways that leads to inflammation and apoptosis, thus resulting in ulceration and further damage inflicted by bacterial colonization on the surface of lesions [6, 7]. Improved irradiation techniques, control of comorbidities, and adequate oral hygiene mitigate the burden of OM [8]. Furthermore, specific prophylactic substances have been proposed, such as mucosal protectors, steroidal and non-steroidal anti-inflammatory and antibiotic agents, or growth-factors, but none of these approaches is considered sufficient to prevent the lesions [1].

Low-power laser irradiation, at present known as photobiomodulation, has been used since the 1980s to control OM [9]. This procedure can reduce pain, severity, and duration of the lesions [10]. Visible or infrared light energy originates intracellular photochemical reactions capable of controlling pain stimuli and in the last instance to stimulate tissue repair [11]. The use of photobiomodulation has steadily increased among dental care providers to oncologic patients due to being readily accessible and easy to use, of a noninvasive nature, and having no serious adverse effects [12, 13]. However, photobiomodulation protocols for the treatment of OM vary widely, leading to difficulties in standardizing its use in clinical settings [14–16]. Moreover, the risk of stimulating the growth of neoplastic or residual neoplastic cells has limited its use to control OM-affected head and neck cancer patients [8].

In order to improve understanding of the clinical effect and risk of protocols for photobiomodulation in the control of radiotherapy-induced OM, the present study compared two doses of laser energy delivered to the oral mucosa of patients undergoing radiotherapy against head and neck cancer, looking for differences in the incidence, onset, severity, or duration of mucositis, as well as in the frequency of tumoral recurrences.

## 2. Materials and Methods

### 2.1. Ethical Considerations

This study was conducted in accordance with the Declaration of Helsinki. It was previously approved by the Institutional Committee for Ethics on Research with Human Subjects (Approval Number: 506.136), and all participants gave their informed consent.

### 2.2. Participants

This prospective study with blinded outcome assessment included all the patients who initiated radiotherapy against head and neck carcinomas (C01 to C06, C09, C10, and C32) from May to July 2015 at the Sector of Oncology of the Hospital of Clinics of Uberlandia, Brazil. Exclusion criteria comprised legal incapacity, previous history of head and neck irradiation, cumulative dose of radiation under 4,000 cGy, symptoms of wasting syndrome, or severe hyposalivation developed before the fourth week of radiotherapy. Irradiation was performed with a 6 mV linear accelerator (Clinac 600C, Varian Medical Systems, CA, USA), in daily doses of 180 cGy five times a week. Data regarding use of tobacco and alcohol, tumor site and staging, and concomitant chemotherapy were recorded for each patient.

### 2.3. Clinical Procedures

All of the patients received dental care before radiotherapy began, including oral prophylaxis, extraction of compromised teeth, restoration of decayed teeth, elimination of periodontal disease, and orientation for the correct use of soft toothbrushes. From the beginning to the end of radiotherapy, the patients were instructed to use an oral suspension with antacid and anesthetic properties as a mouthwash (aluminum hydroxide 60 mg/mL, magnesium hydroxide 20 mg/mL, and oxetacaine 2 mg/mL; Droxaine, Daudt Laboratory, Rio de Janeiro, Brazil) three times a day.

### 2.4. Parameters for Photobiomodulation

This study compared two groups of patients distinguished according to the energy/dose delivered per punctual laser irradiation and thereafter named “0.25 J” and “1.0 J.” Details are provided in Table 1. Patients were randomly allocated to these groups with a previous scheme generated by Excel software (Microsoft Corporation, USA) and were kept unaware of the laser energy being used. The laser was applied by the same operator, at least four-days a week, from the first to the last day of radiotherapy or until resolution of persistent OM lesions. The tip of laser devices was covered with a translucent plastic membrane, and both the patient and operator used goggles. The following anatomical sites received laser irradiation, except when related to the area initially affected by the primary tumor: lip mucosa (four points on each lip), oral mucosa (four points on each side), mobile tongue (three points on each border and one point in the ventral portion), floor of the mouth (one point on each side), and oropharynx (three horizontal points). At each site, the laser beam gently touched the surface at an angle perpendicular to the mucosa. Each laser session lasted approximately ten to fifteen minutes.

### 2.5. Assessment of Oral Mucositis and Tumoral Recurrences

An independent observer (without knowledge of the laser irradiation parameters used for each patient) recorded the occurrence and severity of the lesions according to the World Health Organization (WHO) scale [17] (as shown in Table 2) of OM throughout the entire course of radiotherapy. All of the patients were then followed up at every three months, for two years after the end of the oncologic treatment to register tumoral recurrences. The follow-up consisted of clinical examination, with computerized tomography and biopsy as needed.

### 2.6. Statistical Analysis

Incidence, onset, and severity of OM, as well as the frequency of tumoral recurrence were compared between the groups by means of the chi-square test. The duration of the lesions was analyzed with the unpaired *t*-test. These analyses were performed with the software GraphPad Prism, version 6.03 (GraphPad, San Diego, CA, USA). The level of significance of 5% was established.

## 3. Results

Fifty-eight patients participated in this study. The majority were male (88%), and their age ranged from 30 to 85 years (mean age of 59.5 years). The large majority of the patients were smokers (93.1%) and alcohol abusers or alcoholics (93.1%). Primary carcinomas affected the larynx (39.7%), oropharynx (27.5%), or mouth (32.8%). Most patients harbored advanced carcinomas (79.3% staged III or IV), and half of them presented with regional metastases. None had developed distant metastases. Due to advanced disease, radiotherapy was performed without previous surgical resection in 44 cases (75.9%), of which 31 (70.5%) in adjuvant or neoadjuvant schedule with chemotherapy. Irradiation and chemotherapy were also performed in nine patients after surgical resection of the primary tumor.

**Figure 1:**
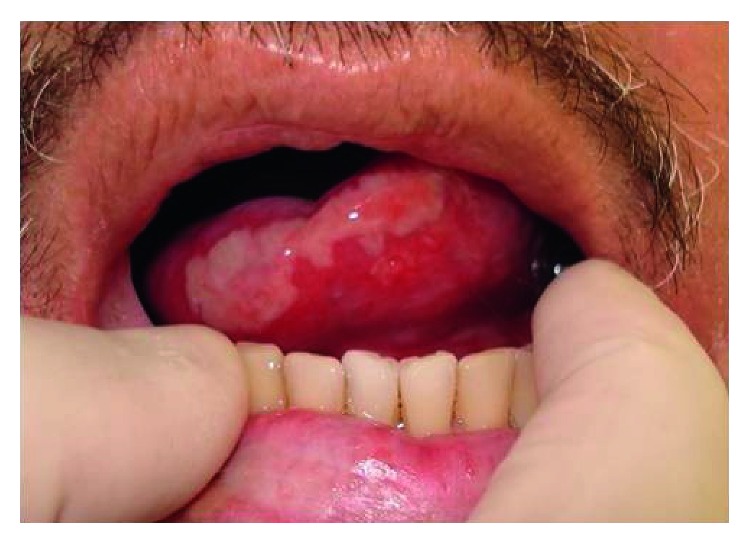
Patient with oral mucositis depicting a large ulcer covered by fibrin pseudomembrane on the left side of the tongue. The lesion was painful, and the patient was unable to eat solid foods (grade 3 on WHO scale [17]).

Thirty patients received 0.25 J of energy per laser irradiation, while 28 patients received 1.0 J of energy. All of the patients tolerated the laser irradiation well. The only complaint was mild to moderate pain when the laser tip was placed in contact with ulcerated lesions. No other adverse effect was found. The comparison of these groups according to the clinical features of the cases is presented in Table 3. As shown in Table 4, OM developed in 42 (72.4%) patients, and it was significantly less frequent in patients receiving 1.0 J of energy. A representative clinical picture of OM is presented in Figure 1. Early onset (in the first week of radiotherapy), severe (grades 3 or 4), or persistent lesions (lasting for two or more weeks) were observed in 20 (34%), 15 (26%), or 40 (69%) patients, respectively. Variation of laser energy did not influence these variables. Tumoral recurrence was found in 14 cases (24%) and did not vary significantly between the groups.

## 4. Discussion

Radiotherapy has gained attention in the treatment of head and neck carcinomas with a view to organ and functional preservation. This progress has been achieved with the development of new schedules for inducing sensitization of neoplastic to radiotherapy cells and improved irradiation technologies [18]. Ionizing radiation exerts its antineoplastic effects through direct induction of DNA double-strand breaks as well as activation of some signaling pathways, both leading to cell death and inflammation [6]. Unfortunately, healthy cells affected by radiation will undergo the same consequences, leading to acute and late-onset adverse effects such as OM, salivary gland dysfunction, radiation caries, taste disturbances, swallowing and speech difficulties, osteoradionecrosis, and disruption of craniofacial development [19]. Furthermore, the interruption of radiotherapy may contribute to the proliferation of residual tumor cells and increase the risk of locoregional recurrences [4]. Therefore, the adverse effects of radiotherapy need to be controlled.

Over 80% of the patients receiving radiotherapy in the head and neck are expected to develop some degree of OM. Nearly 50% of these patients will present severe lesions [2]. Concomitant chemotherapy, hyperfractioned radiotherapy, a great deal of radiation (more than 5.000 cGy), poor oral hygiene, poor nutritional status, lack of antibiotic therapy in the early stage of mucositis, and tobacco smoking have been identified as risk factors for radiation-induced OM [3]. The overall incidence of OM in the present study (74.1%) was mildly better than has previously been reported [2, 12, 20–23], despite the use of high doses of conventional radiotherapy. This was probably due to the effect of intensive preventive measures. This included a magnesium-based oral suspension, which has been empirically prescribed in Brazil as mouthwash for radiotherapy patients, provided some comfort to patients with oral mucositis, in addition to contributing to neutralizing oral pH.

Photobiomodulation of cellular metabolism with an intense but nondestructive light irradiation can be achieved with low-power lasers or light-emitting diodes [11, 12]. This nonpharmacological effect is attributed to the absorption of light energy by endogenous cellular chromophores that start physicochemical biological reactions thus leading to physiological changes [11]. Photobiomodulation increases the synthesis of ATP and reduces the production of reactive oxygen species and proinflammatory cytokines [12–14, 24]. It also stimulates proliferation and migration capacity of fibroblasts [25], collagen synthesis, and angiogenesis, thus favoring tissue repair [26]. In addition, an analgesic effect has been attributed to laser due to depolarization of cellular membranes, inhibition of cyclooxygenase activity, and increased production of endorphins [8, 13]. In the oral cavity, photobiomodulation with laser has been used to control pain related to trauma-associated injury, neuralgia, temporomandibular joint disorders, dentinal hypersensitivity, or ulcerative mucosal lesions, as well as to improve alveolar and soft tissue healing, among other applications [13].

The use of photobiomodulation to control OM was initially reported at the beginning of the 1990s in patients under treatment with fluorouracil [27]. Sooner after, it was applied to reduce severity and duration of radiation-induced OM [9]. Photobiomodulation is able to reduce pain and severity and to postpone the onset of OM [9, 12, 20, 21, 23, 28–30]. Visible, red wavelength (633–685 nm) lasers have been considered useful in preventing OM when less than 2 J/cm^2^ is applied on the mucosa and for therapeutic purposes with a minimal dose of 4 J/cm^2^ [10]. The energy densities used in the present study, corresponding to 6.3 and 33 J/cm^2^, lie within these thresholds.

However, there is no consensus on the parameters for the use of photobiomodulation to control radiation-induced OM. For instance, there is variation in laser devices, schedule according to radiotherapy, wavelength, energy, time, and anatomical sites for irradiation [14–16, 31]. The reduced intensity or very brief use of light will not produce an adequate biologic response, while exceedingly intense or prolonged exposure will inhibit this response [24]. Thus, it is relevant to investigate the most effective combination of irradiation and time. A meta-analysis of randomized clinical trials observed a nonstatistically significant reduction in the risk of OM with higher doses of laser energy when compared with placebo [31]. However, only one previous study indeed compared two protocols of energy output and observed better results with higher energy (3.8 J versus 1.3 J) [22]. The energy outputs of 0.25 J and 1.0 J per irradiation were independently evaluated in different previous studies and were both effective in the reduction of OM when compared with placebo [20, 32, 33]. In the present study, the prophylactic use of 1.0 J of energy per spot instead of 0.25 J resulted in a reduction of nearly 30% in the incidence of radiation-induced OM. This result was mainly derived from a decrease in grades 1, 2, and 4 in patients receiving 1.0 J; however, grade 3 increased. Nevertheless, the relevance of this improvement must be seen with caution since variation in energy output did not influence severity or duration of the lesions.

Finally, there are concerns regarding the use of lasers in areas with previous tumors due to a potential growth stimulating effect on residual neoplastic cells [8]. The enhanced proliferation of neoplastic cell lines after irradiation with low-power lasers support this assumption [34]. On the other hand, experimental evidence has demonstrated that low-power laser did not cause genotoxicity or mutagenicity despite cytotoxicity related to oxidative stress [35]. In our study, the frequency of recurrences was not associated with the energy of laser irradiation. This result must be seen with caution in face of the limited extension of follow-up (two years) but can be taken as an evidence of the safety of photobiomodulation to prevent radiation-induced oral mucositis in cancer patients. In this sense, photobiomodulation to control OM has recently been associated with better prognosis (progression-free survival) for patients with head and neck carcinomas [36].

In conclusion, photobiomodulation with high doses of laser energy (1.0 J versus 0.25 J) produces a small improvement in the prevention of radiotherapy-induced OM and did not significantly increase the risk of neoplastic recurrence.

## Figures and Tables

**Table 1:** Protocols of laser irradiation (adapted from Zecha et al. [11]).

	Protocol	
	Group 0.25 J	Group 1.0 J
Source (laser device)	Indium gallium arsenide phosphide semiconductor laser^∗^	Indium gallium arsenide phosphide semiconductor laser^∗∗^
Wavelength	660 nm (visible red)	660 nm (visible red)
Power	25 mW	100 mW
Beam area	4 mm^2^	3 mm^2^
Energy (radiation)	0.25 J	1.0 J
Time	10 s	10 s
Dosage (fluence or energy density)	6.3 J/cm^2^	33 J/cm^2^
Operating mode	Continuous wave	Continuous wave
Physical relationship to the organ	Intraoral, direct contact with oral mucosa	Intraoral, direct contact with oral mucosa
Schedule	Concomitant to radiotherapy	Concomitant to radiotherapy

^∗^Twin Flex Evolution, MM Optics Ltda, São Carlos, São Paulo, Brazil; ^∗∗^Laser Duo, MM Optics Ltda, São Carlos, São Paulo, Brazil.

**Table 2:** Classification of oral mucositis (adapted from WHO [17]).

Score	Severity	Typical manifestation
0	None	None (no signs or symptoms).
1	Mild	Oral soreness and erythema.
2	Moderate	Oral erythema and ulcers, both solid and liquid diets tolerated.
3	Severe	Oral ulcers, requires liquid diet only.
4	Life-threatening	Oral alimentation not possible.

**Table 3:** Clinical features of the cases.

		Group		*p*
		0.25 J	1.0 J	
Mean age (year)		59.5	59.5	>0.05^∗^
Smokers		29 (96.7%)	26 (89.3%)	>0.05^∗∗^
Alcoholics		28 (93.3%)	26 (89.3%)	>0.05^∗∗^
Primary tumor location	Larynx	13 (43.3%)	10 (35.7%)	>0.05^∗∗^
	Oropharynx	9 (30.0%)	7 (25.0%)	
	Mouth	8 (26.7%)	11 (39.3%)	
Clinical stage of primary tumors	I or II	5 (16.7%)	7 (25.0%)	>0.05^∗∗^
	III or IV	25 (83.3%)	21 (75.0%)	
Association to chemotherapy		23 (76.7%)	18 (64.3%)	>0.05^∗∗^
Tumoral recurrence		8 (26.7%)	6 (21.4%)	>0.05^∗∗^

^∗^ *t*-test; ^∗∗^chi-square test.

**Table 4:** Occurrence of oral mucositis.

Oral mucositis		Group		*p*
		0.25 J	1.0 J	
Incidence	No	4	11	0.04^∗^
	Yes	26	17	
Onset	Early (1st week)	8	12	0.27^∗^
	None or timely (2nd week or after)	22	16	
Duration (weeks)	Mean (±SEM)	2.9 (0.3)	2.5 (0.5)	0.51^∗∗^
Severity	None, grades 1 or 2	24	19	0.37^∗^
	Grades 3 or 4	6	9	

^∗^Chi-square test; ^∗∗^unpaired *t*-test.
